# Massive hemoptysis in intravenous drug users: Case report and review of literature

**DOI:** 10.4103/1817-1737.30363

**Published:** 2007

**Authors:** V. Aiyappan, M. Muthiah

**Affiliations:** Queen Elizabeth Hospital, Kings Lynn, Norfolk, United Kingdom, E-mail: drvinodaiyappan@doctors.org.uk; * *Bassetlaw Hospital, Worksop, S81 0BD*

Dear Sir,

Drug abuse being a rising menace in developing nations, the re-emergence of necrotizing infections in this vulnerable population and its association with massive hemoptysis are issues that need to be addressed.

A 25-year-old intravenous drug user (using heroin) was brought to the Accident and Emergency ward after an episode of massive hemoptysis. He was being treated in the Community for chest infection for the preceding 2 weeks and had been having minor hemoptysis for 3 days. On arrival he was unresponsive. Resuscitation attempts were unsuccessful. Postmortem showed right-sided lung abscess with intraabscess cavity hemorrhage and right-sided empyema. There was no evidence of infective endocarditis/mycotic aneurysms. No organisms were isolated from the abscess.

A 28-year-old intravenous drug user (using heroin) was admitted with history of cough and hemoptysis (100 ml). He was on warfarin for deep vein thrombosis. International Normalized Ratio on admission was 1.6. Physical examination was unremarkable. He underwent computerized tomographic pulmonary angiogram, which showed infective changes in right upper lobe and lower lobe and small thrombus in right upper lobe artery. He was treated with antibiotics and his anticoagulation was continued. His symptoms subsided. Blood culture was negative. Three days later he had massive hemoptysis, and he died. Postmortem showed diffuse dissemination of blood throughout bronchial tree; scarring and bronchiectatic changes, right lower lobe; no evidence of infection/ thromboembolism/ endocarditis. Massive hemoptysis was attributed to bronchiectasis and anticoagulation.

Massive hemoptysis is arbitrarily defined as expectoration of 100 to 600 ml of blood in 24 h.[1] It is a life-threatening condition meriting prompt intervention. In the general population, the commonest causes of massive hemoptysis are bronchiectasis and lung infections.

Intravenous drug users damage their lung by direct effects of the drug itself, by the act of injection or by indirect effects (HIV and other infections). Heroin and cocaine are the main illicit drugs used intravenously. Intravenous heroin has been reported to cause structural changes in the lung, leading to bronchiectasis.[2]

IV drug use related behavior results in increased incidence of pulmonary infections. O'Donnell and Pappas reported an incidence of 23.5% of septic pulmonary emboli in intravenous drug users.[3] Heroin has been reported to alter the immune function by its effect on inducible nitric oxide synthase, and its use may lead to increased susceptibility to infection.[2]

The incidence of massive hemoptysis in intravenous drug users is not known. IV drug use related behavior puts these people at increased risk of severe necrotizing lung infections. This is complicated by associated factors like deep vein thrombosis and anticoagulation. In addition, there is the issue of poor compliance to treatment. Because of the above factors, this group of patients is at high risk of death from massive hemoptysis.
